# Seasonal Dynamics of Trace Elements in Tidal Salt Marsh Soils as Affected by the Flow-Sediment Regulation Regime

**DOI:** 10.1371/journal.pone.0107738

**Published:** 2014-09-12

**Authors:** Junhong Bai, Rong Xiao, Qingqing Zhao, Qiongqiong Lu, Junjing Wang, K. Ramesh Reddy

**Affiliations:** 1 State Key Laboratory of Water Environment Stimulation, School of Environment, Beijing Normal University, Beijing, P. R. China; 2 Wetland Biogeochemistry Laboratory, Soil and Water Science Department, University of Florida, Gainesville, Florida, United States of America; Fudan University, China

## Abstract

Soil profiles were collected in three salt marshes with different plant species (i.e. *Phragmites australis*, *Tamarix chinensis* and *Suaeda salsa*) in the Yellow River Delta (YRD) of China during three seasons (summer and fall of 2007 and the following spring of 2008) after the flow-sediment regulation regime. Total elemental contents of As, Cd, Cu, Pb and Zn were determined using inductively coupled plasma atomic absorption spectrometry to investigate temporal variations in trace elements in soil profiles of the three salt marshes, assess the enrichment levels and ecological risks of these trace elements in three sampling seasons and identify their influencing factors. Trace elements did not change significantly along soil profiles at each site in each sampling season. The highest value for each sampling site was observed in summer and the lowest one in fall. Soils in both *P. australis* and *S. salsa* wetlands tended to have higher trace element levels than those in *T. chinensis* wetland. Compared to other elements, both Cd and As had higher enrichment factors exceeding moderate enrichment levels. However, the toxic unit (TU) values of these trace elements did not exceed probable effect levels. Correlation analysis showed that these trace elements were closely linked to soil properties such as moisture, sulfur, salinity, soil organic matter, soil texture and pH values. Principal component analysis showed that the sampling season affected by the flow-sediment regulation regime was the dominant factor influencing the distribution patterns of these trace elements in soils, and plant community type was another important factor. The findings of this study could contribute to wetland conservation and management in coastal regions affected by the hydrological engineering.

## Introduction

Sediment contamination in rivers, lakes, reservoirs, and wetlands has been widely reported in the developing countries as a result of the intense land use in agricultural and urban environment. Contamination of sediments with trace elements is a major concern [1], [2]. Moreover, these trace elements can be transferred and carried downstream into wetland ecosystems, and accumulate in wetland soils [3], [4]. Wetland soils serve as “source” and “sink” of these chemical pollutants [5]. For example, salt marshes and estuarine sediments can retain these metals as metal sulfides [5], [6]. However, seasonal hydrological changes and water level fluctuation can affect Eh and pH of wetlands, thus resulting in mobilizing trace elements [5], [7].

Several studies on large river delta have focused on source identification of trace elements and organic pollutants in surface sediments [6], [8], [9], [10]. Some studies have shown that heavy metals can be accumulated and retained in wetland soils for a long time period [11], and they would not vary significantly over seasons in salt marshes under natural conditions [12]. The global regulation of rivers and steams by building reservoirs and dams has brought great effects on downstream ecosystems [13]. Recently, Bai et al. [4] have reported that the flow-sediment regulation contributed to trace elements (i.e. Arsenic, Cadmium, and others) accumulation in surface wetland soils of the Yellow River Delta as bed sands can carry and settle these trace elements to the downstream due to strong hydraulic flushing [12], [14]. However,less information is available on the dynamics and fate of these trace elements in estuarine wetlands after the flow- sediment regulation.

The Yellow River Delta (YRD) is one of the most active regions of land-ocean interaction, and a national nature reserve was established to better protect this newly-formed wetland ecosystem and maintain biological diversity [15]. However, with the rapid development of agriculture, fisheries, and the extensive exploitation of the Shengli oilfield (It is the second largest oilfield in China and is mainly located in both sides of the Yellow River Estuary, which was originally built in 1962 and the working area has covered approximately 4.4×10^4^ km^2^), the Yellow River Delta was greatly impacted by the intense human activities, leading to serious wetland degradation [16]. Moreover, the flow-sediment regulation regime has shown a significant influence on trace element accumulation [4] and wetland plant distribution [16] in the Yellow River Delta.

The primary objectives of this study were: (1) to determine the dynamic changes of selected trace elements including As, Cd, Cu, Pb, and Zn in wetland soils covered by different dominant plant species (i.e., *Phragmites australi*s, *Suaeda salsa* and *Tamarix chinensis*) of the Yellow River Delta; (2) to assess enrichment levels of these trace elements and determine their ecological risks using enrichment and toxic indicators; and (3) to identify the relationships among trace elements and other selected soil properties.

## Materials and Methods

### Ethics statement

Our study area is located in the Yellow River Delta wetland nature reserve, which is owned by the Chinese government. We obtained a specific permit from the administration bureau of the Yellow River Delta National Nature Reserve to conduct this study in the reserve. Moreover, our sampling sites were not located in any strictly protected areas and the field studies did not involve endangered or protected species.

### Study area

**Figure 1:**
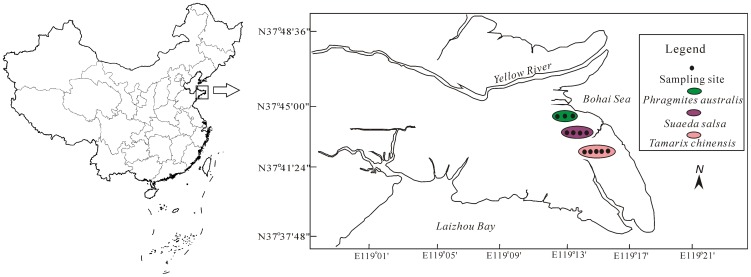
Location map of three sampling sites in the Yellow River Delta.

The study area is located in the Yellow River Delta of China (37°37′48″ to 37°48′36″ N and 119°01′ to 119°21′ E) (Figure 1). It has a temperate, continental monsoon climate with distinct seasons. The annual mean air temperature is 12°C, with 196 frost-free days, and the annual mean precipitation is 552 mm [17]. Soils are characterized as loamy fine sand or coarse sand in all soil layers with clay content of 5–7% and can be classified as aquents, aquolls and fluvaquents [18]. The dominant vegetation in tidal salt marshes include herbaceous plants such as *Phragmites australi*s and *Suaeda salsa* and woody plant namely *Tamarix chinensis* [17]. The vegetation succession in this delta follows the order *Suaeda salsa* → *Tamarix chinensis* → *Phragmites australi*s, and plant distributions are dominantly controlled by soil salinity. The study area was consistently influenced by the flow-sediment regulation of the upstream Xiaolangdi Reservoir from June and July since 2002 [4]. Xiaolangdi Reservoir began storing water in 1999, and considerable siltation occurred in the reservoir after commissioning, with a total sediment trapping of 32.47×10^8^ t from 1997 to 2007 [19]. The flow-sediment regulation of the upstream Xiaolangdi Reservoir has been enforced from June to July since 2002 [17]. For this study, three sampling sites with different dominant plant species (Table 1) were selected. The three sites are approximately 100 m away from each other and they were consistently inundated by tidal seawater (twice every day).

**Table 1:** Ecological characteristics of three sampling sites in different sampling seasons.

Site	Location	Replicates	Sampling time	Plant height (m)	Plant density (m^−2^)
*P. australis* wetland	37°43′35.1″ to 37°43′59″N 119°12′42″ to 119°13′21″E	3	Spring	0.1–0.3	35–50
			Summer	1.8–2.2	230–280
			Fall	1.8–2.2	190–230
*T. chinensis* wetland	37°42′47″ to 37°42′52″N 119°13′49″ to 119°14′15″E	5	Spring	1–1.6	15–30
			Summer	1–2	15–30
			Fall	1–2	15–30
*S. salsa* wetland	37°43′16″ to 37°43′34″N 119°13′36″ to 119°13′53″E	4	Spring	0.05–0.15	100–220
			Summer	0.20–0.40	280–580
			Fall	0.30–0.45	136–520

Note: Spring = April; Summer = August; Fall = November.

### Sample collection and analysis

Soil samples were collected using a soil auger (4.8-cm diam.) from three sampling sites in summer (August, 2007), fall (November, 2007), and spring (April, 2008). Well-developed root systems were observed to depths of 50 cm at *P. australi*s wetland and *T. chinensis* wetland, whereas the root zone was confined to depths of 15–20 cm at *S. salsa* wetland. Therefore, in each sampling season, the top 50 cm soils (sectioned into 0–10, 10–20, 20–30, and 30–50 cm) were collected with three or five replicates in *P. australi*s wetland and *T. chinensis* wetland, and the top 20 cm soils (sectioned into 0–10 and 10–20 cm) were sampled with four replicates in *S. salsa* wetland. A total 120 soil samples were collected, including 36 samples in *P. australi*s wetland, 60 samples in *T. chinensis* wetland, and 24 samples in *S. salsa* wetland. All soil samples were placed in polyethylene bags and brought to the laboratory, where they were air dried at room temperature for three weeks. Some air-dried soils of selected two profiles at each site in each sampling season were used for soil particle size analysis. All the other air-dried soil samples were sieved through a 2-mm nylon sieve to remove coarse debris and then ground with a pestle and mortar until all particles passed a 0.149-mm nylon sieve for the determination of soil chemical properties. Soil bulk density cores were also correspondingly collected using a 184 cm^3^ cylinder from each soil layer of each soil profile, oven dried at 105°C for 24 h, and weighed for the determination of bulk density (BD) and soil moisture.

Plant samples for the aboveground parts (including stems and leaves) were also collected at each soil sampling site in summer. They were placed in paper bags after clean washing and transported to the laboratory. All plant samples were oven dried at 65°C for 48 h, and ground into fine powder for the determination of trace metals. Soil and plant samples were digested with an HClO_4_-HNO_3_-HF mixture in Teflon tubes to analyze total contents of Al, As, Cd, Cu, Pb, and Zn. The solutions of the digested samples were analyzed by inductively coupled plasma atomic absorption spectrometry (ICP-AAS). Quality assurance and quality control were assessed using duplicates, method blanks and standard reference soil materials (GBW07401) and standard reference plant materials (GBW07602) from the Chinese Academy of Measurement Sciences with each batch of samples (1 blank and 1 standard for each 10 samples). The recovery rates of samples spiked with standards ranged from 99 to 106% for soils, and from 82–119% for plants. Soil organic matter (SOM) was measured using dichromate oxidation [20]. Soil pH and salinity were determined in the supernatants of 1∶5 soil and water mixtures using a pH meter or salinity meter. Soil particle size was analyzed using a Laser Particle Size Analyzer (Microtrac Inc., USA).

### Enrichment factor (EF)

Enrichment factor (EF) was selected to evaluate possible anthropogenic inputs of observed sample metals[21]. EF was defined as 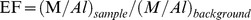, where M_sample_ and M_background_ are the determined contents of targeted elements (e.g., As, Cd, Cu, Pb, and Zn) in soil samples and their background contents, respectively; Al _sample_ and Al _background_ are the determined content s of Al in soil samples and its background content, respectively; Al is used the reference element because it is a conservative lithogenic element presumed not to be enriched due to local contamination [22]. Contamination categories were classified based on EF values: EF<2, deficiency to minimal enrichment; EF = 2–5, moderate enrichment; EF = 5–20, significant enrichment; EF = 20–40, very high enrichment; EF>40, extremely high enrichment [23]. Background values for M_ background_ and Al _background_ in this study were referenced to background contents in the loess materials of the Yellow River [24]. Background contents of 62700 µg g^−1^ for Al, 10.7 µg g^−1^ for As, 0.095 µg g^−1^ for Cd, 21.1 µg g^−1^ for Cu, 21.6 µg g^−1^ for Pb and 64.5 µg g^−1^ for Zn were used.

### Toxic units (TUs)

Toxic units (TUs) are used to normalize the toxicities of various trace elements to allow for the comparison of the relative effects. TUs were defined as the ratios of the observed contents to the probable effect level (PEL) values [25]. PEL values represent the thresholds of chemical contents above which adverse effects are likely to occur. The PEL values for marine and estuarine ecosystem was used in this study (Table 2)[26].

**Table 2:** Summary of physical-chemical properties in the top 20 cm soils at three sampling sites during three sampling seasons.

	Moisture (%)	BD(g cm^−3^)	SOM(g kg^−1^)	Salinity(‰)	pH	Sand (%)	Clay (%)
*P. australis* sites							
Spring	28.8±2.9^a1^	1.4±0.1^a1^	6.9±0.9^a1^	1.5±0.3^a1^	8.4±0.1^a1^	52.1±8.1^a1^	5.2±0.8^a1^
Summer	30.7±4.4^a1^	1.8±0.1^b1^	6.7±1.0^a1^	0.6±0.2^b1^	8.4±0.1^a12^	23.8±4.1^b1^	13.9±2.5^b1^
Fall	28.0±2.0^a1^	1.8±0.0^b1^	8.2±0.6^b1^	0.7±0.1^b1^	8.6±0.1^a1^	55.7±4.5^a1^	5.0±0.2^a1^
*T. chinensis* sites							
Spring	23.6±1.9^a2^	1.5±0.1^a1^	7.0±3.0^a1^	3.4±1.0^a2^	8.3±0.2^a1^	81.1±22.2^a12^	0.7±0.9^a2^
Summer	23.0±1.6^a2^	1.9±0.1^b1^	5.2±1.8^b1^	2.8±0.7^ab2^	8.1±0.2^a1^	61.34±2.8^a2^	4.9±1.6^b2^
Fall	25.0±2.2^a12^	1.8±0.0^b1^	5.9±2.2^b2^	2.0±0.5^b2^	8.6±0.1^b1^	60.7±6.5^a1^	0.3±0.2^b1^
*S. salsa* sites							
Spring	26.2±1.6^ab12^	1.5±0.1^a1^	6.8±1.4^a1^	1.7±0.4^a1^	8.4±0.1^a1^	76.2±9.1^a2^	1.5±1.2^a2^
Summer	29.7±4.0^a1^	1.8±0.1^b1^	6.5±0.5^a1^	0.6±0.4^b1^	8.7±0.3^ab2^	23.12±1.8^b1^	14.2±0.1^b1^
Fall	23.1±2.2^b2^	1.9±0.1^b1^	5.5±1.3^b2^	2.1±1.1^a2^	8.7±0.1^b1^	81.1±3.2^a2^	0.6±0.3^a1^

Spring = April; Summer = August; Fall = November. BD = Bulk density; SOM = Soil organic matter.

a,b different letters represent significant differences between three seasons at each site.

1,2 different numbers represent significant differences between three sites in each season.

### Biological Concentration Factors (BCFs)

Biological concentration factor is the ratio of metal content in the aboveground parts of plants to metal content in surrounding soils, which can more accurately describe the plant's uptake potential than plant metal content [27].

### Statistical analysis

Pearson correlation analysis was performed to determine the relationships between trace elements and selected soil properties and the relationships between Al, trace elements, and sand and clay contents in selected soil profiles. Principal component analysis (PCA) was used to discriminate soil samples with similar or different contamination patterns and identify their influencing factors. Analysis of variance (ANOVA) analysis was implemented to analyze the differences in trace elements between sampling sites and seasons. Differences were considered to be significant at *p*<0.05. Statistical analysis was performed using the SPSS 16.0 and Canoco 4.5 software packages for Windows.

## Results and Discussion

### Soil characteristics in three salt marshes

Selected physical-chemical properties in the top 20 cm soils of three salt marshes of the YRD are summarized in Table 2. No significant differences in soil moisture among three seasons in each sampling sites due to consistent tidal flooding condition. However, Soils in *P. australi*s wetland contained higher moisture contents in spring and summer compared to *T. chinensis* wetland due to less sand contents in *P. australi*s wetland (*p*<0.05; Table 2). Bulk density at each sampling site of the three was lower in spring than in summer and fall (*p*<0.05), but there were no significant differences among three sites in each season. With the exception of higher SOM level in *P. australi*s wetland than those in *T. chinensis* and *S. salsa* wetlands in fall (*p*<0.05), no significant differences were observed among three sites in summer and spring. Compared to other seasons, higher SOM content appeared in *P. australi*s wetland in fall, while it was higher in *T. chinensis* wetland in spring (*p*<0.05), which was likely caused by plant litter inputs and decomposition [5]. All soil samples of three sites exhibited higher salinities in spring than in summer or fall (*p*<0.05), which was associated with the freshwater inputs from the flow-sediment regulation [4]. Moreover, *T. chinensis* wetland showed higher soil salinity (*p*<0.05) than *P. australi*s wetland or *S. salsa* wetland in three seasons as *T. chinensis* wetland was further away from the river channel (Figure 1). However, no significant differences in pH values were observed among three wetlands in spring and fall except that *T. chinensis* wetland had lower pH values (*p*<0.05) than *S. salsa* wetland in summer. Both *T. chinensis* and *S. salsa* wetlands showed higher pH values (*p*<0.05) in fall compared to spring and summer, which might be associated with weaker soil respiration in fall as the dissolved CO_2_ in overlaying water could decrease pH values [28]. All sampling sites showed higher clay contents in summer than in spring and fall and sand contents were also lower in both *P. australi*s and *S. salsa* wetlands in summer (*p*<0.05; Table 2). This indicated that the flow and sediment regulation in summer brought more clay and silt contents to the tidal salt marshes [4]. However, no significant differences in sand or clay contents were observed between spring and fall, which was associated with tidal seawater erosion [4]. Moreover, soils in *P. australi*s and *S. salsa* wetlands contained more clay contents and less sand contents in summer compared to *T. chinensis* wetland (*p*<0.05). This might be caused by the fact that *T. chinensis* wetland is located at the farther distances from the Yellow River Chanel (Figure 1). In both spring and fall, *P. australi*s wetland had higher clay contents than *T. chinensis* and *S. salsa* wetlands.

### Spatial and temporal variations in trace elements in marsh soils

**Figure 2:**
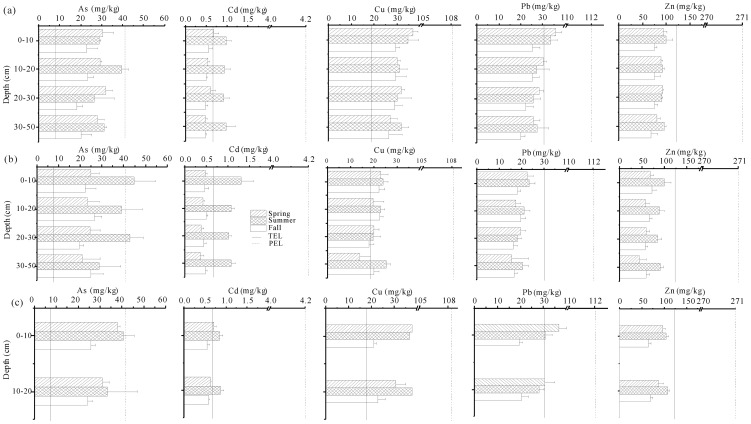
Profile distributions of As and heavy metals in marsh soils with *P. australis* (a), *T. chinensis* (b) and *S. salsa* (c) in three sampling seasons.

Profile distributions of these trace elements in three seasons are shown in Figure 2. Generally, trace element contents exhibited a decreasing tendency with depth in each season except for As, Cd and Zn in *P. australi*s wetland and Cd, Cu and Zn in *S. salsa* wetland in summer. Prusty et al. [29] also reported that trace elements such as As, Cu, Pb, and Zn decreased with increasing depths in wetland ecosystems. This was greatly associated with plant cycling which lead to trace elements upwards movement through plant litters and return to surface soils [29], [30]. However, clear seasonal changes in the contents of these trace elements were observed along soil profiles (Figure 2). Among three seasons, most soil profiles of three sampling sites exhibited the highest levels of As, Cd and Zn in summer, followed by spring, whereas the lowest levels in fall. As for Cu and Pb, lower levels were also observed in fall compared to summer and spring. The average contents of As, Cd and Zn in the top 20 cm soils in *P. australi*s and *T. chinensis* wetlands were significantly higher in summer than in fall (*p*<0.05; Table 3). Similarly, much higher average contents of these trace elements in the top 20 cm soils were also observed in *S. salsa* wetland in summer compared to fall (*p*<0.05; Table 3). This is inconsistent with the results by Roychoudhury [12], who presented that trace metal contents in surface soils were relatively lower in summer than in winter due to higher wetland plant uptake and standing stocks of metals in summer. Additionally, Ehlken and Kirchner [31] reported that higher water contents, lower salinities and bulk densities increased the potential mobility of metals in soil solutions. However, all trace elements generally reached their higher levels in summer in this study, although all soils contained lower salinities and higher water contents in three sampling sites after the flow-sediment regulation, and then fell to the lowest levels in fall. Therefore, the higher trace element contents observed in summer might be attributed to exogenous inputs of trace metals (e.g., water and sediment inputs from the flow-sediment regulation) [4], [32]. The increased plant uptake of trace elements in the late fall could explain the lower trace element contents in these marsh soils in this season, as plants possess efficient root-to-shoot translocation systems that are activated at the end of the growing season [33].

**Table 3:** Mean contents of As and heavy metals in the top 20 cm soils of three sampling sites during the whole sampling period (mg kg^−1^ dry weight).

	As	Cd	Cu	Pb	Zn
*P. australis* sites					
Summer	35.8±2.4^a1^	0.9±0.1^a1^	30.7±5.5^a1^	28.56±5.3^ab1^	95.1±11.0^a1^
Fall	23.0±4.8^b1^	0.5±0.1^b12^	29.2±1.5^a1^	25.27±1.3^b1^	74.7±5.7^b1^
Spring	29.8±3.5^c1^	0.6±0.1^b1^	33.5±4.3^a1^	32.9±2.4^ac1^	91.2±10.4^a1^
*T. chinensis* sites					
Summer	41.9±3.2^a2^	1.2±0.2^a2^	23.0±2.3^a2^	22.4±2.8^a2^	93.9±13.1^a2^
Fall	24.7±3.4^b1^	0.5±0.0^b2^	22.1±1.4^a2^	19.4±2.8^a2^	72.0±10.7^b1^
Spring	24.1±4.5^b2^	0.4±0.0^b2^	21.4±1.6^a2^	20.1±1.8^a2^	62.5±4.5^c2^
*S. salsa* sites					
Summer	40.5±6.4^a2^	0.9±0.1^a1^	37.7±1.7^a1^	29.9±1.9^a1^	106.3±3.8^a2^
Fall	25.2±1.8^b1^	0.6±0.0^b1^	22.0±2.1^b2^	19.9±2.4^b2^	66. 9±6.2^b1^
Spring	34.5±1.4^c1^	0.7±0.0^bc3^	34.5±3.7^a1^	33.7±4.5^a1^	92.1±9.5^c1^
Sediment Quality Criteria of China*					
Class I	20	0.5	35	60	150
Class II	65	1.5	100	130	350
Class III	93	5	200	250	600
SQGs†					
TEL	7.2	0.68	18.7	30.2	124.0
PEL	41.6	4.2	108.2	112.2	271.0

a,b different letters represent significant differences between three seasons at each site.

1,2 different numbers represent significant differences between three sites in each season.

* National Standard of P.R. China [37].

† SQGs: Sediment Quality Guidelines for marine ecosystem; TEL: threshold effect level; PEL: probable effect level [23].

**Figure 3:**
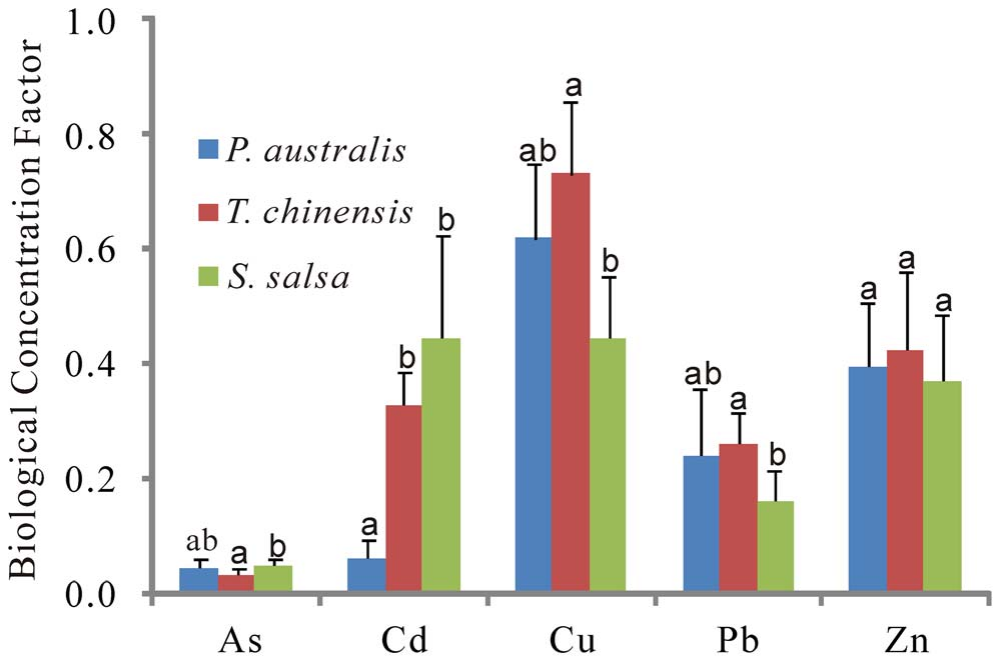
Biological concentration factors of the aboveground parts of plants in each site. ^abc^ Different letters on the error bars represent significant differences (*p*<0.05).

**Figure 4:**
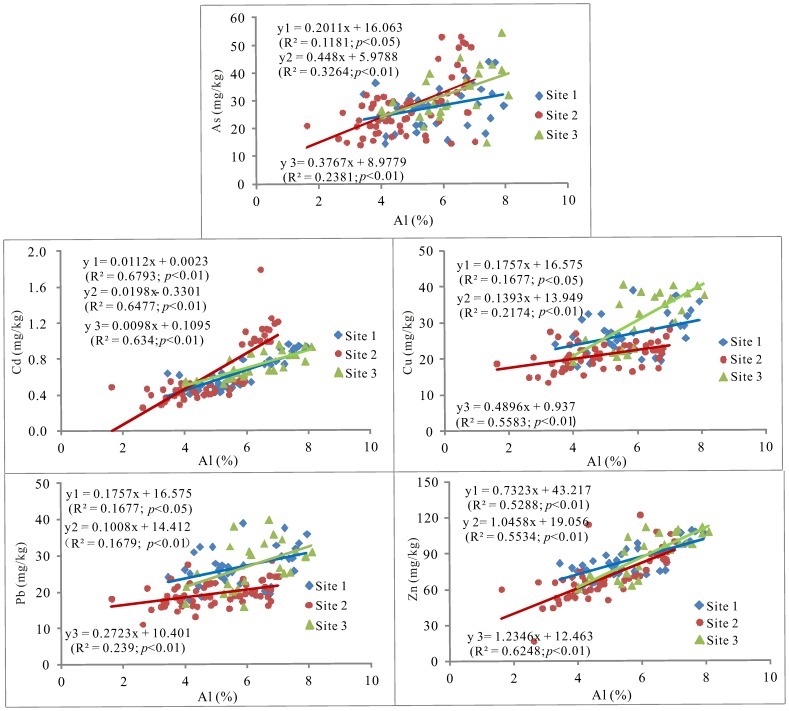
Relationships between the contents of aluminum (%) and trace elements (mg/kg) in all soil samples in the Yellow River Delta.

Both Cu and Pb contents in soil profiles were generally lower in *T. chinensis* wetland than those in *P. australi*s and *S. salsa* wetlands in spring and summer, whereas higher levels of As and Cd in soil profiles were observed in *T. chinensis* wetland in summer (Figure 2). Table 3 also showed that the average contents of trace elements in the top 20 cm soils were significantly higher in *P. australi*s and *S. salsa* wetlands in spring compared to *T. chinensis* wetland (*p*<0.05). Meanwhile, higher levels of Cu and Pb in *P. australi*s wetland and higher Cd contents in *S. salsa* wetland were observed in fall compared to other sites (*p*<0.05). In summer, *P. australi*s and *S. salsa* wetlands exhibited greatly higher Cu and Pb contents than *T. chinensis* wetland (*p*<0.05, Table 3). The differences in Cu and Pb between sites or sampling seasons were most likely related to SOM, sand and clay contents (Tables 4 and 5). Additionally, lower Cu and Pb levels in the top 20 cm soils in *T. chinensis* wetland in summer compared to *S. salsa* wetland might be caused by higher plant uptake as the aboveground parts of *T. chinensis* had higher BCFs values for both Cu and Pb compared to *S. salsa* (*p*<0.05; Figure 3). However, it is difficult to explain much higher BCFs values of *T. chinensis* for Cd with higher soil Cd levels (*p*<0.05; Table 2) than *P. australi*s. Therefore, the variations in trace element levels among the three sampling sites might be influenced by the multiple environmental factors including plant communities, soil properties, and water levels etc. [34], [35], [36]. As shown in Figure 4, all trace elements generally showed similar band-type distribution patterns, indicating higher levels of these trace elements in *P. australi*s and *S. salsa* wetlands higher compared to *T. chinensis* wetland except for some soil samples with higher levels of As and Cd. These trace elements increased linearly with increasing Al content at each sampling site, which implies that these trace elements and Al might have common origin.

**Table 4:** Relationships between soil texture and Al and heavy metals in typical profiles from three sampling sites in three sampling seasons.

	Al	As	Cd	Cu	Pb	Zn
Sand	−0.743**	−0.306*	−0.259*	−0.805**	−0.753**	−0.121
Clay	0.758**	0.305*	0.263*	0.850**	0.756**	0.276*

* represents significant correlation at the level of *p* < 0.05;

** represents significant correlation at the level of *p* < 0.01.

n = 60.

**Table 5:** Correlation matrix among trace elements and selected soil properties for three sampling sites.

	Moisture	Bd	SOM	Salinity	pH	S	Al	As	Cd	Cu	Pb	Zn
Moisture	1.000											
Bd	0.210*	1.000										
SOM	−0.006	−0.189*	1.000									
Salinity	0.143	−0.179	−0.151	1.000								
pH	0.238**	0.201*	−0.038	−0.376**	1.000							
S	0.034	−0.262**	0.478**	0.099	0.014	1.000						
Al	−0.028	0.212*	0.144	−0.265**	−0.141	0.140	1.000					
As	−0.097	0.020	0.037	0.009	−0.295**	0.137	0.507**	1.000				
Cd	−0.082	0.277**	0.131	−0.087	−0.346**	0.062	0.745**	0.618**	1.000			
Cu	−0.139	−0.193*	0.496**	−0.297**	0.110	0.455**	0.617**	0.343**	0.377**	1.000		
Pb	−0.147	−0.348**	0.440**	−0.203*	0.021	0.455**	0.529**	0.371**	0.347**	0.883**	1.000	
Zn	−0.155	0.021	0.278**	−0.237**	−0.101	0.262**	0.783**	0.571**	0.723**	0.772**	0.726**	1.000

* represents significant correlation at the level of *p* < 0.05;

** represents significant correlation at the level of *p* < 0.01.

n = 144.

### Assessment of trace element pollution by sediment quality guidelines (SQGs)

The extent of trace element pollution was assessed by comparing element contents in marsh soils to the sediment quality criteria developed by the Chinese Marine Sediment Quality Criteria (GB 18668-2002) [37] and the sediment quality guidelines (SQGs)[23] (Table 3). The Chinese Marine Sediment Quality Criteria indicated that approximately 85–95% of all collected soil samples in three sampling sites during three seasons exceeded the Class I criteria (suitable for fisheries and natural waters) but fell within the scope of Class II criteria (used for industrial and tourism sites) for As and Cd, and all samples were below the Class I criteria for Cu, Pb and Zn (Table 3). This supplies that most soil samples in this region have been moderately polluted by As and Cd. As shown in Figure 2, the SQGs also indicated that almost all soil samples along soil profiles at three sites fell within the range between the threshold effect levels (TELs) and probable effect levels (PELs) for As and Cu in each of the three seasons, even some samples of three sites exceed PELs in summer. Moreover, all soil profile samples at each site exceeded TELs in summer, whereas they were below TELs in both spring and fall. However, approximately 85% of soil profile samples in this region didn't exceed TELs for Pb in three seasons, and almost all soil profile samples at each site exhibited much lower Zn contents than TELs in each season. This implies higher potential risks of As, Cu and Cd in soil profiles of this region. Therefore, it is still required to control heavy metal pollution (i.e., As, Cu and Cd) in this region, as higher trace elements may make it more difficult to restore the degraded living habitat [36].

### Enrichment factors of trace elements in marsh soils

**Figure 5:**
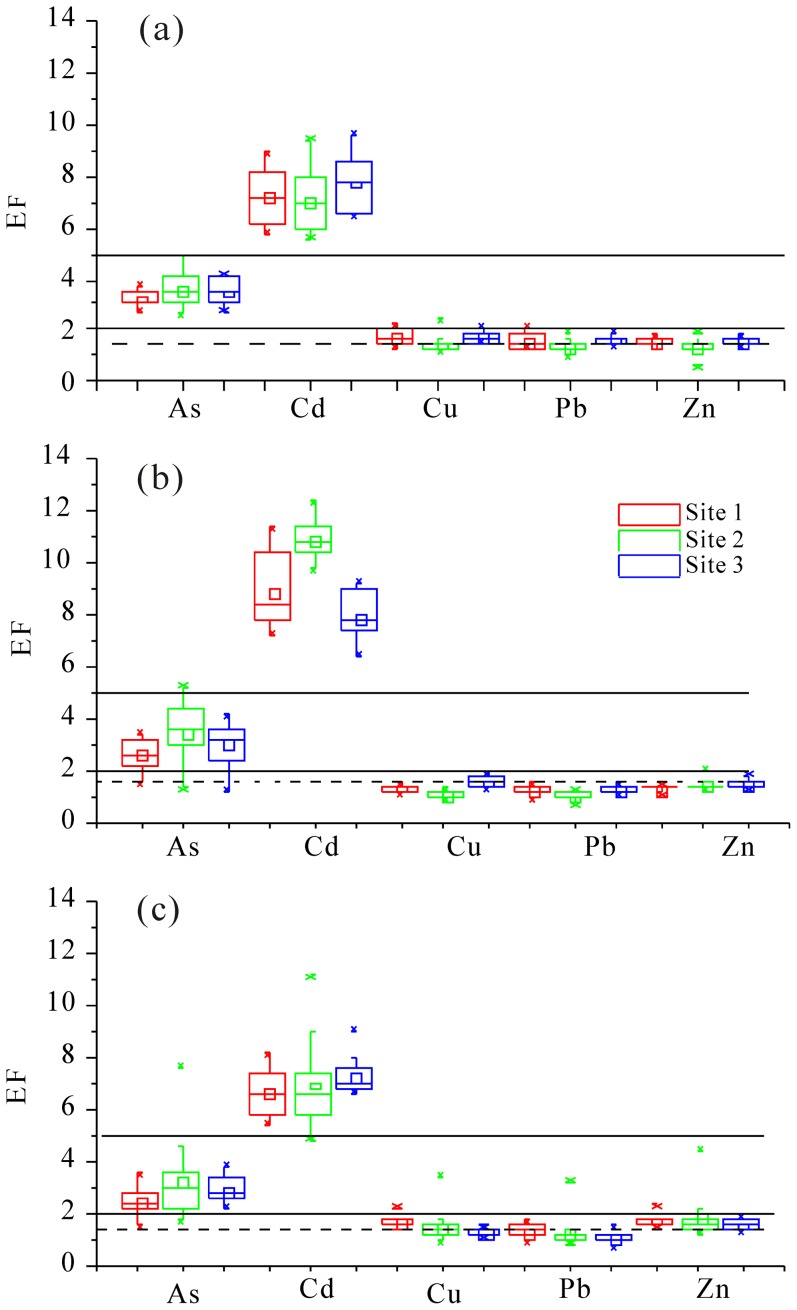
Box-plots of enrichment factors of trace elements in all soil samples of each site of the three in three seasons (a) Spring; (b) Summer; (c) Fall. Straight lines represent the EF = 2 or 5; dash line represents EF = 1.5.

Although the contents of trace elements were lower in the YRD than in most large rivers and estuaries [4], trace element pollution in the YRD is increasingly serious due to continuous disturbances caused by human activities and sediment movements [36], [38]. Trace element contents in the upstream loess were used as indicators to determine the enrichment levels for trace elements in the soils. Figure 5 shows the proportions of enrichment levels in marsh soils at three sampling sites in each season. Cd levels in all soil samples were at the significant enrichment level in three seasons. As levels in almost 95% of soil samples were at a moderate enrichment level. Comparatively, more than 90% of soil samples showed minimal enrichment levels of Cu, Pb and Zn in three sampling sites in each season. Both *P. australi*s and *T. chinensis* wetlands showed higher enrichment levels of Cd and lower enrichment of Cu, Pb, and Zn in summer than in both spring and fall, which was associated with higher BCFs of Cu, Pb and Zn and lower BCFs of As and Cd at both sites (Figure 3). Moreover, almost all EF values of As and Cd and more than 50% of EF values of Cu, Pb and Zn for all soils in three seasons exceeded 1.5, indicating that a significant portion of metal is delivered from non-crustal materials, or non-natural weathering processes, so anthropogenic sources might be an important contributor [39]. Bai et al. [4] also presented that the flow-sediment regulation could elevate the levels of trace metals.

### Ecotoxicity assessments of trace elements

**Figure 6:**
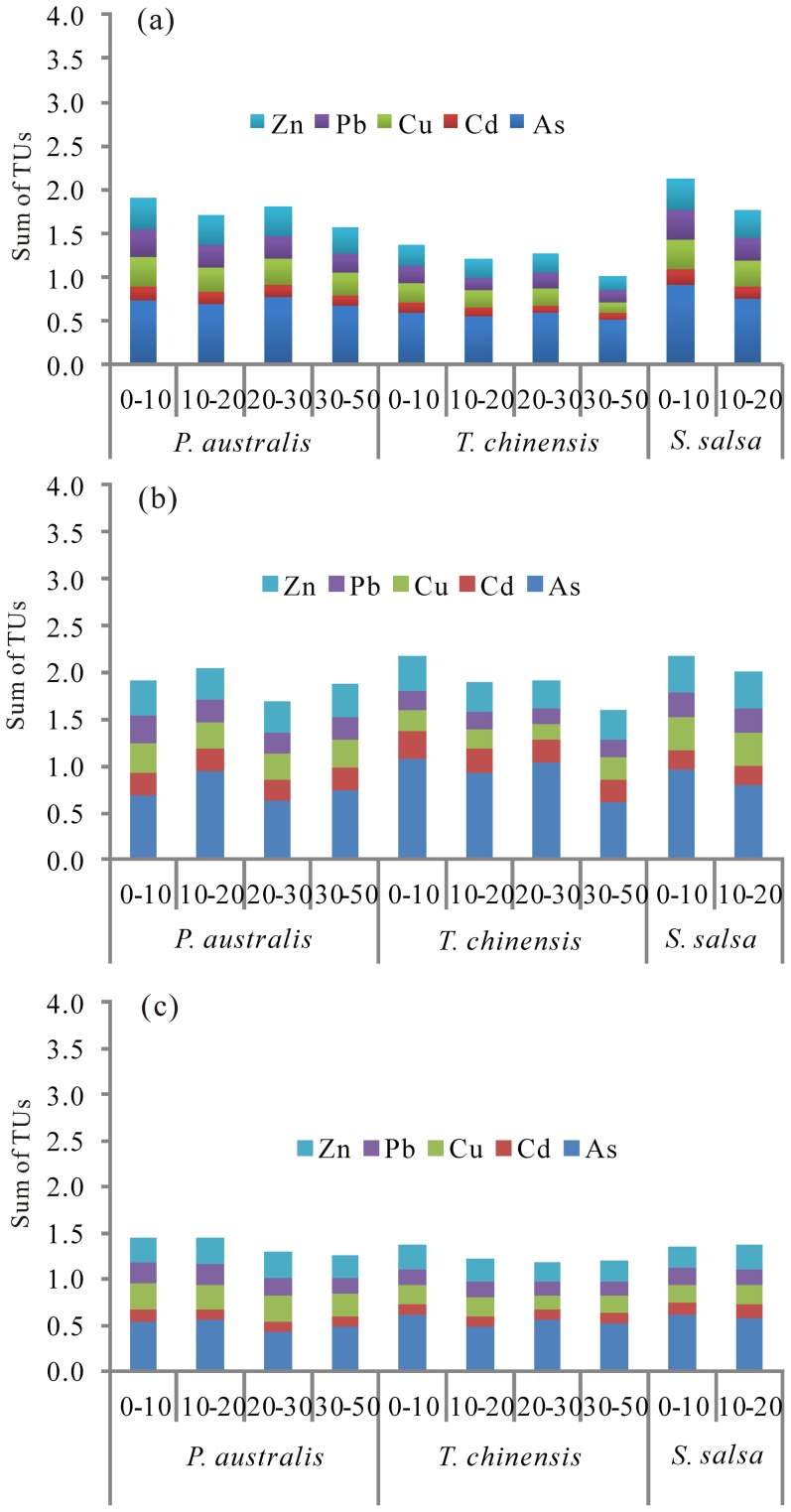
The sum of the toxic units (∑TUs) and toxic contribution of each metal at each soil layer of three sampling sites in three seasons (a) Spring; (b) Summer; (c) Fall.

The potential acute toxicities of contaminants in soil samples can be estimated using the toxic unit (TU) index, which is the ratio of the determined content to the probable effect level (PEL) [26]. The TUs, the sum of toxic units (∑TUs) and relative contributions of trace elements at each soil layer in the YRD are illustrated in Figure 6. The mean TUs value of each element at three sites decreased in the order of As > Zn > Cu > Pb > Cd in each of three seasons. As and Cd in all soil samples had much higher TUs in summer compared to spring and fall. Generally, the ∑TUs decreased with depth along soil profiles. The ∑TUs for surface soils (0–10 cm) in *S. salsa* wetland in spring or in *T. chinensis* wetland in summer were much higher than those for all the other soil samples of the three sites, but neither exceeded the moderate toxicity level (∑TUs  =  4;) [25]. The order of the ∑TUs in the top 20 cm soils of three sampling sites in spring was *S. salsa* wetland > *P. australi*s wetland >*T. chinensis* wetland. Additionally, the ∑TUs for all soil layers at the three sites reached higher levels in summer than in fall and spring. The ∑TUs for each layer were higher in *P. australi*s and *S. salsa* wetlands than *T. chinensis* wetland in spring, whereas they exhibited similar ∑TUs levels at three sites in summer and fall.

The values of the ∑TUs increased from spring to summer and then decreased to their lowest levels in fall. This corresponded to the temporal variations in trace element contaminations during the three sampling seasons which was closely related to the input flow containing trace elements from upstream during the flow-sediment regulation period from June to July. Tixier et al. [40] presented that the storm water exhibited the seasonally varying toxicity of trace elements with high toxicity in spring and recovery in fall, which was caused by the Terraview–Willow field storm water management facility. The ∑TUs for all soil layers of the three sampling sites were less than 4, suggesting low ecotoxicity in the current YRD soils. This is consistent with the result by Gao and Chen [41], who presented that the surface sediments showed no or low ecotoxicity of trace elements in the coastal Bohai Bay. However, As manifested the highest contribution ratios (ranging from 24.4 to 53.7%) to the ∑TUs at three sites in each of the three seasons compared to other trace elements (Figure 6). Therefore, the soils were ‘As toxic’ (with TUs for As greater than 0.5), indicating that As might have greatly negative effects and ecological risks on some or all of the environmental components [42].

### Relationships between trace elements and soil physico-chemical properties

The correlation analysis between trace elements in the marsh soils of each sampling site was performed to assess possible co-contamination from similar sources. As shown in Table 4, significantly positive correlations among all trace elements (i.e., As, Cd, Pb, Cu, and Zn) were observed, suggesting that these trace elements in marsh soils might originate from common source. Figure 4 also exhibited that the linear relationships between Al and these trace elements at each sampling site, which implies that these trace elements and Al were greatly associated with the upstream loess materials with higher Al contents of the Yellow River (62700 µg g^−1^) [24]. Moreover, Al and these trace elements were significantly correlated with sand and clay contents (Table 4), indicating that higher clay contents in August (Table 2) would contribute to these trace element accumulation. Therefore, the upstream flow-sediment regulation in June could take main responsibility for the accumulation of these trace elements in the delta in the studied period due to no other pollution source inputs in the study area. This is also supported by the result of Bai et al. [4], who presented that the upstream flow-sediment regulation contributed to elevating trace element contents in surface soils in this delta.

Table 5 showed the negatively linear correlations between salinity and Al, Cu, Pb and Zn in the study area (*p*<0.01). This is in agreement with the results presented by Du Laing et al. [43], indicating increasing salinity might enhance the mobility of these metals in soils, as the chloro complex formation and the substitution of Ca^2+^ and Mg^2+^ in the exchange positions could promote the mobility of heavy metals [44]. Additionally, Manousaki et al. [45] found that salinity could favor metal accumulation in shoots of salt cedars, thus decreasing soil metal contents. Bulk densities exhibited negative correlations with Cu and Pb in all soils, implying that lower bulk densities in saturated substances with higher SOM favored the accumulation of both metals. Numerous studies reported that SOM and soil pH played important roles in determining the fates of heavy metals in soils [11], [46]. SOM can act as a major sink for heavy metals due to its strong complexing capacity for metallic contaminants [47]. In this study, SOM showed a positive linear relationship with Cu, Pb and Zn (*p*<0.01; Table 5). Most researchers presented that subalkaline environments could decrease the mobility of metals and increase the ability of soil to stabilize metals [11], [36], [48]. However, in this study, soil pH values were negatively correlated with As and Cd (*p*<0.01; Table 5). This was most likely due to plant uptake or leaching as alkalization will increase metal leaching at higher pH ranges (8–10) [46], [49]. Sulfur exhibited significantly positive correlations with Cu, Pb and Zn (*p*<0.01) due to the deposition of metal sulphates (i.e., CuS, PbS and ZnS) under anaerobic conditions [4]. Moreover, Kumpiene et al. [46] reported that the presence of one contaminant (e.g., Cu or Pb) can decrease the stabilization efficiency of another contaminant (e.g., Zn) due to competition for sorption sites. Therefore, heavy metals levels in the marsh soils could be impacted by such factors as moisture, salinity, SOM, pH values, sulfur and the interactions between trace elements.

**Figure 7:**
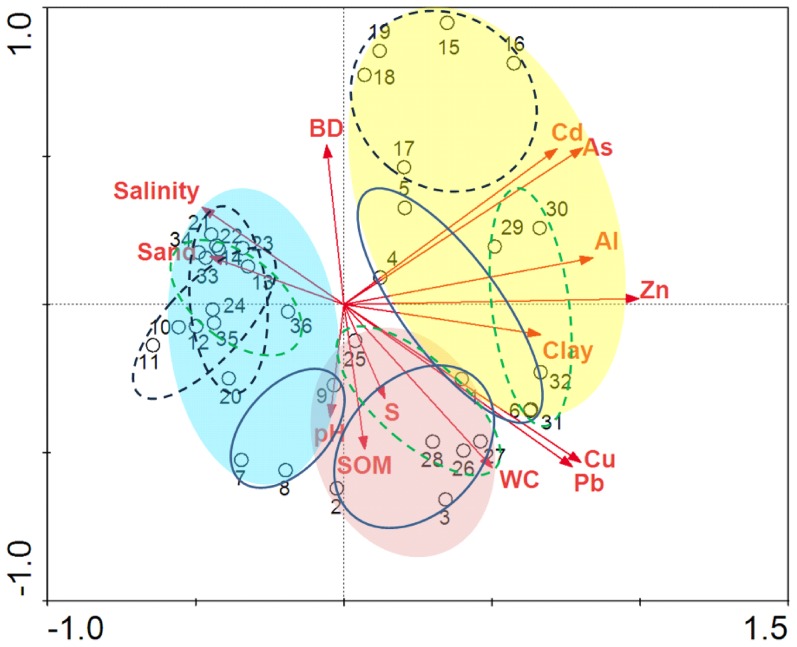
Ordination plots of the PCA results for surface soil samples. The direction of an arrow indicates the steepest increase in the variable, and the length indicates the strength relative to other variables. S, sulfur; BD, bulk density; SOM, soil organic matter; WC, moisture. No. 1, 2, 3 represent three replicates of *P. australis* wetland in spring; No. 4, 5, 6 represent three replicates of *P. australis* wetland in summer; No. 7, 8, 9 represent three replicates of *P. australis* wetland in fall; No. 10, 11, 12, 13, 14 represent five replicates of *T. chinensis* wetland in spring; No. 15, 16, 17, 18, 19 represent five replicates of *T. chinensis* wetland in summer; No. 20, 21, 22, 23, 24 represent five replicates of *T. chinensis* wetland in fall; No. 25, 26, 27, 28 represent four replicates of *S. salsa* wetland in spring; No. 29, 30, 31, 32 represent four replicates of *S. salsa* wetland in summer; No. 33, 34, 35, 36 represent four replicates of *S. salsa* wetland in fall.

To assess the relationships between spatial distribution patterns of trace elements and several environmental factors (including 5 soil parameters), PCA was implemented to characterize the variations in trace element contents among sampling sites. The factor loading scores in the biplot (Figure 7) exhibited a clear separation amongst *P. australi*s, *T. chinensis* and *S. salsa* wetlands and amongst spring, summer, and autumn along the first principal component (PC) accounting for 75.1% and the second PC accounting for 16.0% of the variance. As shown in Figure 7, three main groups can be clearly identified. The red plate contained the soils collected in spring, the yellow plate contained the soils collected in summer, and the blue plate included the soils collected in fall. It appears that the sampling seasons play an important role in shaping the spatial distribution patterns of As and heavy metals in marsh soils. However, the soils in *T. chinensis* wetland in spring did not fall into the red plate but entered or attached to the blue plate, suggesting that the distribution patterns of trace elements in those soils are more similar to those in *T. chinensis* and *S. salsa* wetlands in fall than those in *P. australi*s and *S. salsa* wetlands in spring.

PCA exhibited that the strongest determinants of the distribution patterns of trace elements in spring were soil moisture, S, SOM and pH value, while the strongest influencing factors in fall were soil salinity and soil texture. Obviously, salinity, water content and soil texture controlled the distribution patterns of trace elements in *T. chinensis* and *S. salsa* wetlands, while SOM, S and soil pH values were the dominant factor in *P. australi*s wetland. All trace elements with the longest arrows in the summer group testified the highest accumulations of trace elements in summer, especially in *P. australi*s and *S. salsa* wetlands with herbaceous plants. The results obtained from the PCA were consistent with the results derived from the correlation analysis.

Although spatial distribution patterns of trace elements were dominantly controlled by sampling seasons due to the flow-sediment regulation, the effects of plant species on their distributions in soils could not be ignored due to different plant accumulation capabilities for different species (Figure 3). As shown in Figure 7, in the same season plate, sampling sites with the same plant community were clustered together. This was because plant roots can significantly affect plants' abilities to transfer metals [50], [51], as they employed a variety of ways to alleviate the stresses from root zone pollution through increasing the rate of root exudation as a result of losses of membrane integrity or of breakdowns in normal cell metabolism [52], [53]. The fixation and stabilization of trace elements by plants originating from physiological characteristics (e.g., root exudation in response to underground stress) were different between herbaceous plants (*P. australi*s and *S. salsa*)) and woody plant (*T. chinensis*). Additionally, eclogical characteristics of plants (e.g., plant density) might also affect the responses of plants to trace elements, especially under the interactive impacts of water and salt in the tidal salt marshes. This implies that marsh soils with different plant communities might maintain different capabilities for immobilizing trace elements at the same pollution level (Figure 3). Further studies are needed to investigate the impacts of land cover changes on these trace elements.

## Conclusions

Obvious spatial and seasonal changes in trace elements in wetland soils in the intertidal salt marshes were observed. The higher trace element contents (especially As and Cd) in summer compared to spring and fall suggested the contribution of the flow-sediment regulation (from June to July) to pollutants from upper reaches deposition and accumulation in the delta and higher self-purifying capabilities of these salt marshes. This implies that these tidal salt marshes can alleviate the effects of the upstream flow-sediment regulation. Higher trace element contents in these soils were closely correlated to soil properties such as soil moisture, salinity, sulfur, SOM, soil texture and pH value. It is noted that sampling seasons had dominant impacts on the spatial distribution patterns and ecotoxicity of trace elements in soils in this study. Therefore, seasonal changes in trace element contamination should be taken into account in tidal wetlands especially in tidal wetlands with great hydrological fluctuations. We also observed that marsh soils with herbaceous plants (*P. australi*s and *S. salsa*) had higher trace element accumulation than woody plant (*T. chinensis*). Although all soil samples showed lower ecotoxicity, higher ecotoxicity of As in each of the three seasons should attract our concerns in this region. Moreover,it is important to continuously monitor these trace elements in coastal wetland soils to identify the ecological and environmental effects of hydrological engineering. The findings of this study could contribute to wetland conservation and management in coastal regions affected by the hydrological engineering. Further studies of the final fate and transport of these trace elements and plant uptake mechanism under water and salt stress in coastal wetlands will greatly contribute to trace element pollution control in coastal region.
